# Preliminary Data of the First Year of Newborn Screening for Metachromatic Leukodystrophy (MLD) in Lombardy

**DOI:** 10.3390/ijns12030049

**Published:** 2026-06-30

**Authors:** Alessandra Vasco, Andrea Meta, Clarissa Berardo, Davide Camerlengo, Francesca Fumagalli, Davide Tonduti, Iris Fiamingo, Cristina Montrasio, Diana Postorivo, Manuela Rizzetto, Sabrina Fede, MLD-NBS Study Group, Roberto Bozic, Francisco Ferron, Valeria Calbi, Gianvincenzo Zuccotti, Alessandro Aiuti, Giancarlo la Marca, Stephana Carelli, Cristina Cereda

**Affiliations:** 1 Center of Newborn Screening, Functional Genomics and Rare Diseases, Department of Pediatrics, Buzzi Children’s Hospital, 20154 Milan, Italy; alessandra.vasco@aorncaserta.it (A.V.); andrea.meta@asst-fbf-sacco.it (A.M.); berardo.clarissa@asst-fbf-sacco.it (C.B.); davide.camerlengo@asst-fbf-sacco.it (D.C.); iris.fiamingo@gmail.com (I.F.); cristina.montrasio@asst-fbf-sacco.it (C.M.); diana.postorivo@asst-fbf-sacco.it (D.P.); manuela.rizzetto@asst-fbf-sacco.it (M.R.); sabrina.fede@asst-fbf-sacco.it (S.F.); stephana.carelli@guest.unimi.it (S.C.); 2 Pediatric Research Center “Romeo ed Enrica Invernizzi”, Department of Biomedical and Clinical Sciences, University of Milan, 20157 Milan, Italy; gianvincenzo.zuccotti@unimi.it; 3 San Raffaele Telethon Institute for Gene Therapy (SR-TIGET), 20132 Milan, Italy; fumagalli.francesca@hsr.it (F.F.); calbi.valeria@hsr.it (V.C.); aiuti.alessandro@hsr.it (A.A.); 4 Pediatric Immuno-Hematology Unit, IRCCS Ospedale San Raffaele, 20132 Milan, Italy; 5 Unit of Neurology, IRCCS Ospedale San Raffaele Milan, 20132 Milan, Italy; 6 Unit of Pediatric Neurology, C.O.A.L.A (Center for Diagnosis and Treatment of Leukodystrophies), V. Buzzi Children’s Hospital, 20154 Milan, Italy; davide.tonduti@asst-fbf-sacco.it; 7 Neuroscience Research Center, Department of Biomedical and Clinical Sciences, University of Milan, 20157 Milan, Italy; 8 Revvity SpA—Bastioni di Porta Nuova, 20121 Milan, Italy; roberto.bozic@revvity.com (R.B.); francisco.ferron@revvity.com (F.F.); 9 Medical School, Vita-Salute San Raffaele University, 20132 Milan, Italy; 10 Newborn Screening, Clinical Biochemistry and Clinical Pharmacy Laboratory, Meyer Children’s Hospital IRCCS, 50134 Florence, Italy; giancarlo.lamarca@meyer.it; 11 Department of Experimental and Clinical Biomedical Sciences “Mario Serio”, University of Florence, 50134 Florence, Italy; 12 Department of Biomedical and Clinical Sciences, University of Milan, 20157 Milan, Italy

**Keywords:** metachromatic leukodystrophy (MLD), lysosomal storage disorder, tandem mass spectrometry, newborn screening, sulfatides

## Abstract

Metachromatic leukodystrophy (MLD) is a rare, autosomal recessive lysosomal storage disorder caused by a deficiency of the enzyme arylsulfatase A (ARSA, MIM #250100), which leads to progressive demyelination in the central and peripheral nervous systems, resulting in severe neurodegeneration and premature death. With the recent approval of the first ex vivo gene therapy for MLD, newborn screening (NBS) programs have begun to implement pilot studies for early detection, as identifying affected individuals during the pre-symptomatic therapeutic window is crucial. We present the analytical workflow and the NBS algorithm developed during the first year of MLD screening in Lombardy. The screening strategy comprised a first-tier test (1TT) using mass spectrometry to quantify sulfatide concentrations, a second-tier test (2TT) measuring ARSA enzymatic activity, and a third-tier test (3TT) based on whole-exome sequencing. A total of 16,130 newborns were screened for MLD. Of these, 6.41% required retesting (1TT), 1.53% underwent duplicate 2TT, and 0.36% proceeded to 3TT. No neonates were recalled, and no cases of MLD were identified through the screening program. The first-year implementation of MLD NBS in Lombardy demonstrates that a mass spectrometry-based sulfatide assay combined with a multi-tiered screening algorithm is feasible and reliable. Incorporating genetic analysis alongside expanded validation of additional sulfatide species may enhance specificity, reduce false positives, and facilitate timely identification of infants affected by MLD.

## 1. Introduction

Metachromatic leukodystrophy (MLD) is a rare, autosomal recessive lysosomal storage disorder caused by a deficiency of the enzyme arylsulfatase A (ARSA, MIM #250100), which results in the accumulation of sulfatides in neural and non-neural tissues. This biochemical defect leads to progressive demyelination in the central and peripheral nervous systems, causing severe neurodegeneration and premature death [1,2]. MLD has an estimated incidence of 1 in 40,000 to 170,000 live births, with significant variability among populations [3].

MLD is categorized into three main forms based on age at symptom onset: (i) late-infantile, with onset before 30 months of age; (ii) juvenile, with onset between 2.5 and 16 years, further subdivided into early juvenile (onset 2.5–7 years, typically associated with earlier manifestation and more rapid progression) and late juvenile (onset 7–16 years, generally characterized by later presentation and comparatively slower progression); and (iii) adult, with onset after 16 years of age [2]. The late-infantile form is the most common and rapidly progressive, often presenting with developmental milestone delays, gait disturbances, hypotonia, and loss of motor function. As the disease progresses, patients develop spasticity, seizures, and cognitive decline, leading to a vegetative state and death, typically within a few years from symptom onset [4,5]. Juvenile and adult forms are more heterogeneous in presentation, often starting with behavioral or cognitive changes, psychiatric symptoms, and motor decline that progress at a slower rate [6,7].

The *ARSA* gene is located on chromosome 22q13.33, and it encodes the ARSA enzyme. More than 300 pathogenic variants have been identified, including point mutations, small intragenic deletions/insertions, exon or whole-gene deletions/duplications [8,9]. Some mutations result in a complete loss of enzyme activity (0-type ARSA-MLD alleles), while others allow for residual activity (R-type ARSA-MLD alleles), influencing disease severity and progression. Approximately 90–95% of patients with MLD harbor point mutations or small insertions/deletions in the *ARSA* gene, present in either the homozygous or compound-heterozygous state, whereas large rearrangements have been reported in less than 1% of patients [10]. A distinct class of *ARSA* variants, known as pseudodeficiency alleles (ARSA-PD), comprises common polymorphisms that reduce ARSA activity to below-average levels but do not cause MLD, even when present biallelically or in compound heterozygosity with a pathogenic *ARSA* allele [11].

Although the project is specifically designed to perform NBS for MLD, defects in *PSAP* and *SUMF1* were also considered in the differential diagnosis [10]. Indeed, *PSAP* mutations result in saposin B deficiency, leading to impaired ARSA function despite normal *ARSA* sequencing, while *SUMF1* variants cause multiple sulfatase deficiency with overlapping clinical features.

Hematopoietic stem cell transplantation (HSCT) has shown modest benefit in juvenile- and adult-onset cases, particularly when administered in the presymptomatic or early symptomatic phase [12]. Very recently, a significant milestone in the treatment of MLD was the approval of atidarsagene autotemcelthe first ex vivo gene therapy for MLD available in Europe, the United Kingdom and the United States [13,14]. This therapy is indicated for children with late-infantile or early juvenile MLD who are pre-symptomatic or for early juvenile patients in the very early stages of the disease. The therapy involves harvesting autologous hematopoietic stem cells, transducing them ex vivo with a lentiviral vector carrying a functional copy of the *ARSA* gene, and re-infusing the modified cells into the patient after myeloablative conditioning. Clinical trials have demonstrated that this therapy prevented or significantly delayed the onset of neurological symptoms in treated patients, thanks to the sustained ARSA activity achieved [14]. Treated children have shown preserved cognitive and motor development compared to natural history controls, with follow-up data extending up to 12 years. The median follow-up was 6.76 years (range, 0.64–12.19 years) [14,15]. These advances highlight the importance of early diagnosis, which remains a major challenge due to the rapid progression of the disease and subtle initial symptoms. However, due to the absence of clinical symptoms at birth and the rarity of the disease, most patients are diagnosed only after the onset of irreversible neurological damage [3]. In recent years, newborn screening (NBS) programs have begun to incorporate pilot studies for MLD, leveraging tandem mass spectrometry to detect elevated sulfatide levels in dried blood spot (DBS) and to measure ARSA enzyme activity [16,17]. When coupled with confirmatory genetic testing, this approach holds promise for identifying affected infants before symptom onset, enabling timely access to potentially life-saving therapies [18,19].

As more jurisdictions evaluate the inclusion of MLD in NBS panels, ethical, logistical, and economic considerations will shape implementation. Nonetheless, the synergy between early diagnosis and curative gene therapy represents a paradigm shift in the management of this devastating disease.

In July 2024, a pilot project for NBS of MLD started in the Lombardy region (Italy), involving full-term newborns (≥37 weeks) with normal body weight (≥2500 g) and parental consent to participate in the study. The test is carried out on DBS samples collected at 48–72 h after birth and involves the evaluation of sulfatides and ARSA activity markers, followed by whole exome sequencing (WES).

Here we present the data collected in the first year of the Lombardy region pilot study, with a focus on validation of analytical methods, development of the screening algorithm, and confirmation of the accuracy of the screening algorithm.

## 2. Materials and Methods

### 2.1. Project Study and Data Collection

The study was conducted at the Center of Newborn Screening, Functional Genomics and Rare Diseases at Vittore Buzzi Children’s Hospital and was approved by the LOMBARDIA 1 Territorial Ethics Committee at a meeting held 6 December 2023 (CET143-2023). DBS samples were collected using the standard heel-prick method between 48–72 h of life at 24 newborn centers participating in the project in the Lombardy region. Inclusion criteria were newborns at term (≥37 gestational weeks) and of normal weight (≥2500 g) from parents who consented to participate in the project study. The prospective study started newborn enrollment on 15 July 2024, with the objective of screening a total of 100,000 infants, in accordance with national guidelines in Italy (Ministerial Decree of 13 October, https://www.gazzettaufficiale.it/eli/id/2016/11/15/16A08059/sg, accessed on 19 June 2025) on the same DBS of mandatory NBS. Nine patients previously diagnosed at the Pediatric Immuno-Hematology Unit, IRCCS Ospedale San Raffaele (Milan, Italy), were included as positive controls to evaluate the performance of the first-tier test (1TT) and second-tier test (2TT) within the screening protocol. Specifically, six samples from affected patients older than 48–72 h of life were newly collected as new DBS on Guthrie cards, after informed consent, and processed immediately after collection under the same analytical and pre-analytical conditions routinely adopted for NBS in order to assess ARSA activity at 2TT. Additionally, three archived NBS Guthrie cards from the MLD-diagnosed infants, stored at the Center for Newborn Screening, were retrieved after informed consent for the use of residual material. These archived neonatal DBS samples were used to establish positive values for the 1TT sulfatide assay. The anamnestic and clinical characteristics of all nine MLD patients included as positive controls are reported in Table 1.

### 2.2. Sulfatide Determination (First-Tier Test; 1TT)

Sulfatides were extracted from a 3.2 mm DBS punch, which contains approximately 3.2 μL of sample [20], as previously described by Hong et al. [21]. The determination of sulfatides in DBS samples was performed using an ultra-high-performance liquid chromatography—tandem mass spectrometry (UPLC-MS/MS) method.

The UPLC method was performed using an Acquity UPLC HSS T3 1.8 μm 2.1 mm × 50 mm (Waters Corp, Milford, MA, USA) column coupled to a pre-column Acquity HSS T3 1.8 μm VANGUARD (Waters Corp, Milford, MA, USA) at 45 °C on a UPLC-40 liquid chromatograph (Shimadzu Corp., Kyoto, Japan). The aqueous phase was composed of acetonitrile and water (50:50 *v*/*v*), whereas the organic phase consisted of isopropanol and acetonitrile (80:20 *v*/*v*); all were LC-MS grade. In both phases, formic acid at a concentration of 0.1% *v/v* was added.

The MS/MS method was performed on a Sciex 6500-QTrap^®^ (SCIEX, Toronto, ON, Canada) mass spectrometer. The mass method was set up in multiple reaction monitoring (MRM) detection mode combined with negative mode electrospray ionization (ESI). MRM transitions, mass and ion source parameters were optimized by infusion of available standards.

**Figure 1:**
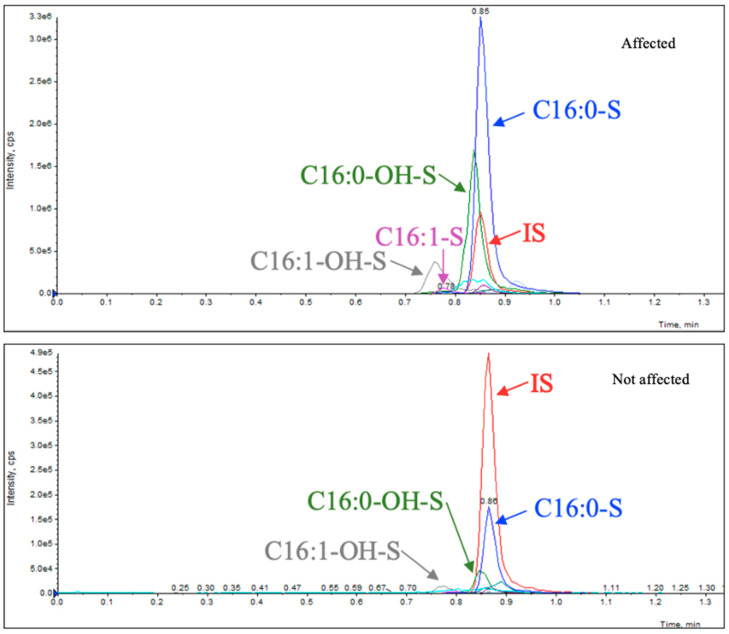
Chromatogram of sulfatides from an MLD-affected neonate (non-neonatal infant, upper) and an unaffected neonate (48–72 h, lower). The *X*-axis shows time (minutes), and the *Y*-axis intensity (counts per second (CPS)). Graph color code: blue, C16:0-S; green, C16:0-OH-S; purple, C16:1-S; gray, C16:1-OH-S; red, C16:0 IS. IS = internal standard.

C16:0-Sulfatide (C16:0-S), C16:0-OH-Sulfatide (C16:0-OH-S), C16:1-Sulfatide (C16:1-S) and C16:1-OH-Sulfatide (C16:1-OH-S) were investigated (Figure 1).

The C16:1OH-Speak exhibited a moderately broadened chromatographic profile, a feature commonly observed for hydroxylated sulfatide species due to their amphipathic structure and stronger interaction with the stationary phase. Nevertheless, peak resolution, retention time stability, and signal reproducibility remained within acceptable analytical limits throughout the study.

To ensure consistent chromatographic performance, daily preventive maintenance of the analytical column was systematically performed, including column flushing with strong organic solvents, re-equilibration of the stationary phase, monitoring of backpressure stability, and verification of system suitability parameters prior to each analytical batch. These procedures minimized carryover effects and maintained the robustness and reproducibility of the LC-MS/MS method.

The semi-quantification of the molecules was carried out by the isotope dilution method, using the same internal standard (IS) for each sulfatide, which corresponds to a C16:0-S marked with 5 deuterium atoms (D_5_-C16:0-S) (Revvity, Waltham, MA, USA). The concentration value of each sulfatide was calculated by multiplying the peak-area ratio (which corresponds to the ratio between the area of the sulfatide and the IS area) of each molecule by the molar concentration of D_5_-C16:0-S IS.

Starting in May 2025, a revised IS solution was incorporated into the routine assay. In addition to D_5_-C16:0-S, the new formulation also includes D_13_-C16:1-OH-S (C16:1-OH-S IS) (Revvity, Waltham, MA, USA). The method for the C16:1-OH-S molecule was optimized using D_13_-C16:1-OH-S as the standard. Although the IS solution contains both sulfatides, quantification is currently performed exclusively using the C16:0-S IS, whereas C16:1-OH-S IS has remained under evaluation for potential future integration into the screening workflow.

To determine the analytical precision of C16:0-S and C16:1-OH-S within each run, three levels of quality control materials (low, medium, and high) (Revvity, Waltham, MA, USA) were analyzed at both the beginning and the end of each plate sequence.

#### 2.2.1. Validation Procedure

The validation process was performed exclusively for the C16:0-S molecule, following the protocols established by the Clinical and Laboratory Standards Institute (CLSI C62 guideline on Liquid Chromatography–Mass Spectrometry Methods) and the European Medicines Agency (ICH guideline M10 on bioanalytical method validation and study sample analysis).

C16:0-S home-made controls were prepared using a 36% albumin solution in phosphate-buffered saline, which exhibits viscosity and protein concentration comparable to whole blood. This solution can be dispensed and absorbed onto the same filter paper used in NBS procedures [22,23].

Trueness, precision, and linearity were assessed through multiple replicates of controls. Trueness was evaluated by calculating percent bias, precision by the coefficient of variation, and linearity by linear regression analysis.

The limit of detection (LOD) and limit of quantitation (LOQ) were estimated using the standard error values derived from the linear regression of repeated measurements at low C16:0-S concentrations. The limit of blank (LOB) was determined by analyzing 60 replicates of samples with C16:0-S concentrations below the LOD.

Matrix effect and recovery were evaluated by comparing three C16:0-S calibration curves—two prepared in matrix and one in solvent.

Carry-over was assessed by injecting a blank sample immediately after a high-concentration C16:0-S sample.

#### 2.2.2. Sulfatide Test Reference Population

Before the initiation of the pilot study, DBS samples obtained from 5000 presumed unaffected, full-term neonates with normal birth weight were analyzed to establish reference population values. All samples were derived from residual DBS material for which parental consent for research use had been obtained.

The 97.5th percentile of the reference distribution was adopted as the cut-off value for 1TT, whereas the 99.0th percentile was applied as the cut-off value of the retested samples to perform the 2TT. Although this threshold generated a relatively higher number of retests, we considered this acceptable during the exploratory phase of method implementation in order to avoid the potential exclusion of subjects with low but clinically relevant biomarker elevations.

### 2.3. ARSA Enzyme Activity (Second-Tier Test; 2TT)

The 2TT consisted of measuring ARSA enzymatic activity in two distinct spots obtained from the same DBS on which the 1TT had been performed.

ARSA enzyme extraction consisted of an initial 4-h extraction phase, followed by purification using Microcon 30K filter columns (Merck Millipore, Milan, Italy) to concentrate and desalt the eluate, and a final 16 h incubation of the purified enzyme with its substrate at 37 °C [24]. In normal subjects, the ARSA enzyme can convert the substrate C18:0-Sulfatide into the reaction product C18:0-galactosylceramide. The product has been determined by UPLC-MS/MS using the synthetic substrate D_3_-C18:Sulfatide and C18:0-galactosylceramide IS marked with 7 deuterium atoms (D_7_-C18:0- galactosylceramide) (Enfanos, Seattle, WA, USA).

**Figure 2:**
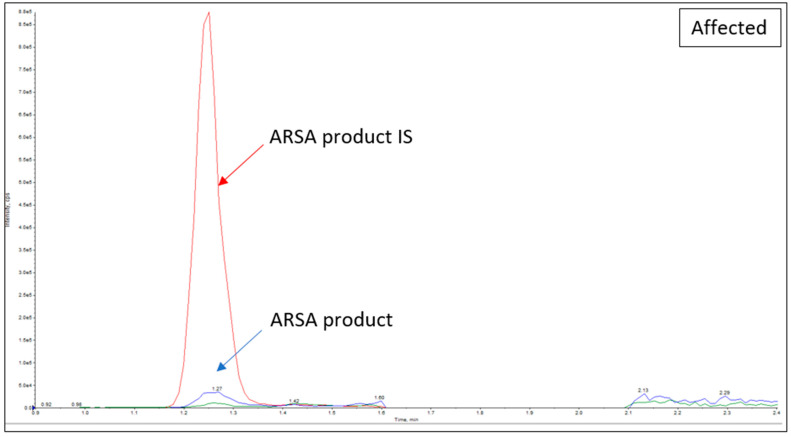

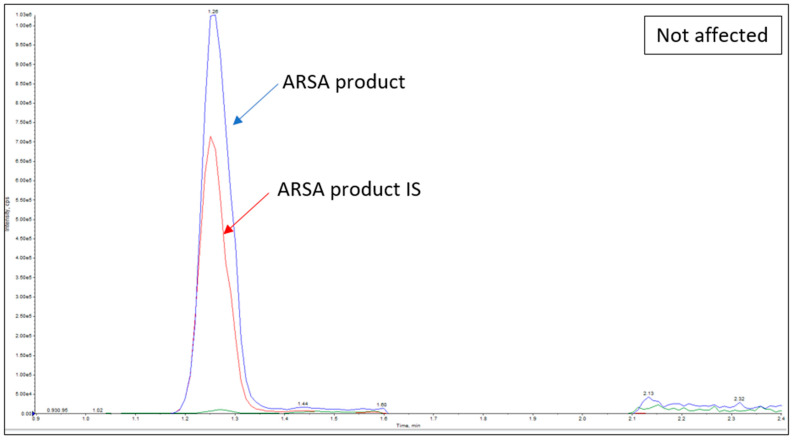
Chromatogram of the ARSA-product reaction of an MLD-affected subject (**upper**) and an unaffected neonate (**lower**). The blue peak, which corresponds to the ARSA reaction product, is present only in the lower chromatogram. The X-axis shows time (minutes), and the Y-axis intensity (counts per second (CPS)). Graph color code: blue, ARSA product (D_3_-C18:0-galactosylceramide); red, ARSA product IS (D_7_-C18:0-galactosylceramide); green, ARSA substrate (D_3_-C18:S).

Chromatographic separation was performed using a CSH Fluoro Phenyl 1.7 µm 2.1 µm × 50 mm (Waters Corp, Milford, MA, USA) column coupled to a UPLC CSH Fluoro-Phenyl 1.7 µm VANGUARD (Waters Corp, Milford, MA, USA) pre-column. The temperature of the column oven was kept at 30 °C and the flow rate was set to 0.7 mL/min. Mobile phase A consisted of acetonitrile LCMS grade and ultrapure water (50/50 *v*/*v*), whereas mobile phase B was isopropanol and acetonitrile LCMS grade at 50/50 *v*/*v*. Both mobile phases were supplemented with 0.1% formic acid of LCMS grade. The gradient started with 20% mobile phase B, gradually increasing to 100% in 1.8 min and kept at 100% B for 1 min. ARSA product and ARSA product IS were eluted at 1.24 min (Figure 2). From time 1.6 to 2 min, the flow was diverted to waste by using the diverter valve built-in on the instrument to minimize the introduction of dirtiness in the mass spectrometer and avoid contamination.

The mass spectrometer was operated in MRM mode using positive electrospray ionization mode (ESI+). MRM transitions, mass and ion source parameters have been optimized by infusion of an available standard (Enfanos, Seattle, WA, USA) (Figure 2).

Each analytical session included a negative control prepared from an adult blood sample and a positive control derived from a patient with a confirmed diagnosis of MLD, followed at San Raffaele Hospital in the context of a natural history clinical trial, who consented to provide blood samples for research purposes.

#### ARSA Assay Activity Reference Population

In the absence of robust data on ARSA activity in newborns affected by MLD identified through NBS, reference ranges expressed as the percentage of enzymatic activity relative to presumed healthy individuals were adopted. To define the most appropriate reference population for the diagnostic workflow, 145 full-term neonates with normal birth weight were selected. All samples were derived from residual DBS material for which parental consent for research use had been obtained.

The reference population was evaluated by estimating the mean values of the two DBS measurements in the cohort. The cut-off was set at the 20th percentile of the ARSA activity average distribution, corresponding to a residual enzymatic activity of 40% of the median value observed.

### 2.4. Genetic Analysis (Third-Tier Test; 3TT)

Genetic analysis was performed using WES with in silico gene panel analysis. Genomic DNA was extracted from DBS using the Chemagic 360 instrument (Revvity, Waltham, MA, USA). DNA quantity and integrity were estimated using the Qubit instrument (ThermoFisher, Waltham, MA, USA) and the 4200 TapeStation system (Agilent Technologies, Santa Clara, CA, USA). Libraries were prepared with “Illumina DNA Prep with Exome 2.5 Enrichment” (Illumina, San Diego, CA, USA) and “Twist Exome 2.0 plus Comprehensive Exome Spike-in” (Twist Bioscience, San Francisco, CA, USA) and sequenced on NextSeq2000 (Illumina, San Diego, CA, USA). Reads were aligned to the NCBI human reference genome build GRCh38. Variant calling and annotation were performed using eVAI software v 3.10 (enGenome, Pavia, Italy) with a virtual panel of 3 genes: *ARSA* (NM_000487.6), the disease-causing gene, *PSAP* (NM_002778.4) and *SUMF1* (NM_182760.4). Therefore, these genes were additionally included in the analysis for research purposes, using residual DBS material, subject to prior parental consent for the use of leftover samples in research.

Variants were assessed using major reference databases (e.g., VarSome, ClinVar, Human Gene Mutation Database) together with the published literature. Variants were also assessed for their frequency in the general population, as reported within public databases (e.g., https://gnomad.broadinstitute.org/, dbSNP), and for their predicted effect. The identified variants were classified according to the American College of Medical Genomics guidelines [25]. Homozygous or compound heterozygous samples for class 3 (VUS—Variant of Uncertain Significance), 4 (LP—Likely Pathogenic), or 5 (P—Pathogenic) variants in the disease-causing gene were considered positive.

### 2.5. MLD Screening Workflow

The samples were classified as non-negative if the concentration of C16:0-S and/or the sum of the 4 sulfatides exceeded the cut-off at the 97.5th percentile at 1TT and underwent a 1TT retest in duplicate on the same DBS using two different spots. In the cases in which the retesting confirmed values above the 99th percentile, the 2TT was performed on two other different DBS from the same Guthrie card. The 2TT was considered positive when at least one of the following criteria was met: (i) both DBS sample spots showed ARSA activity below the established cut-off; (ii) the mean ARSA activity calculated from the two DBS punches was below the cut-off. If 2TT was positive, WES was performed on the same DBS.

In the event of a positive genetic test result, the newborn would be referred to the reference center for rare diseases competent for MLD of the San Raffaele Hospital (Milan, Italy) where the child would be clinically managed and would undergo diagnostic tests to confirm the diagnosis. The diagnostic protocol would include measurement of ARSA activity in leukocytes from whole blood and quantification of urinary sulfatides in case of identification of VUS at the genetic test. Confirmatory genetic analysis would be performed on peripheral blood by the clinical reference center responsible for patient management to confirm the variants previously identified on DBS and to assess the potential compound heterozygous status through family segregation analysis.

**Figure 3:**
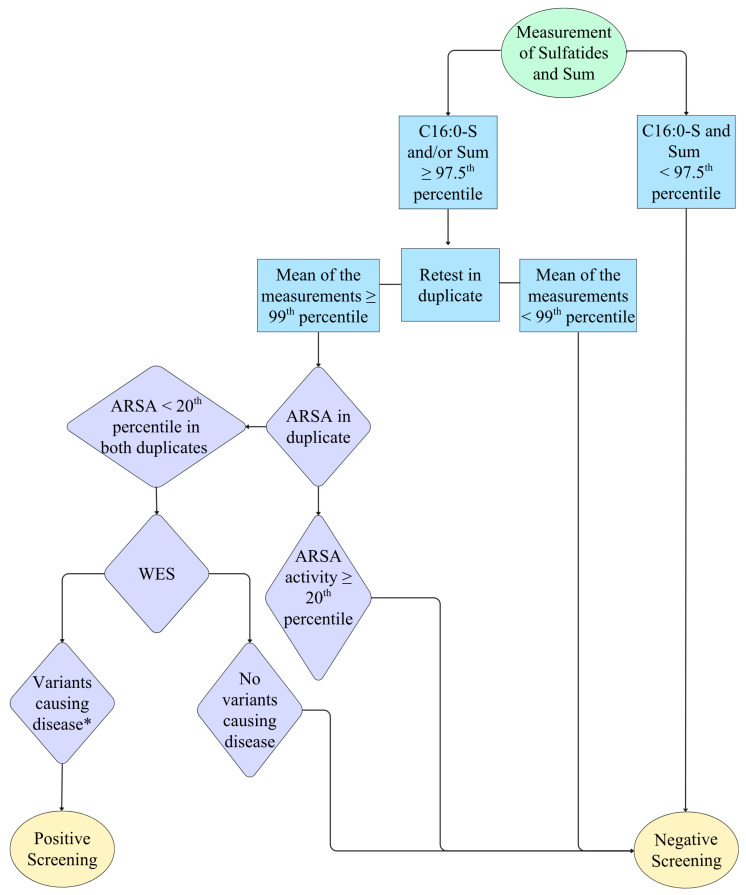
The flowchart represents the algorithm used for newborn screening of MLD. * Homozygous or compound heterozygous samples for VUS, LP or P variants in the disease-causing gene were considered positive.

If the diagnosis is confirmed, the patient would be referred to the available treatment options, after appropriate counseling (Figure 3).

## 3. Results

### 3.1. Validation of the Sulfatide Analysis Procedure

The method satisfied acceptability criteria for all parameters (trueness, precision, and linearity) defined by current guidelines (±15%) for the quantification of C16:0-S.

The calculated LOB, LOD and LOQ were 0.029 nM, 1.58 nM, and 5.69 nM respectively. The matrix effect was determined to be 105%, while the recovery was 79%. Carry-over, assessed in the 10 blank replicates analyzed, yielded a mean concentration of 1.57 nM, corresponding to less than 20% of the LOQ, thus complying with guideline requirements.

### 3.2. Determination of 1TT Cut-Off

A total of 5000 DBS samples, analyzed for the mandatory neonatal screening, were anonymously selected and processed for sulfatide 1TT analysis. All sulfatides were quantified using the C16:0-S IS. Cut-off values for each individual sulfatide marker, as well as for the summed sulfatide parameter, were determined independently. Specifically, for each analyte, quartile distribution analysis was performed, statistical outliers were excluded, and percentile-based thresholds were subsequently calculated on the filtered dataset. Consequently, the cut-off value established for the summed sulfatide parameter does not correspond to the arithmetic sum of the individual marker cut-offs, since it was derived from an independent statistical distribution analysis of the total sulfatide values. Table 2 shows the respective 97.5th and 99th percentile cut-offs.

Following the implementation of the new IS formulation incorporating C16:0-S IS and C16:1-OH IS molecules, the reference intervals were re-established based on the analysis of an additional cohort of 5000 newborns undergoing routine screening, which served as the reference population. The statistical analysis was conducted in the same manner as for the previous population. C16:1-OH-S was quantified using the C16:1-OH-S IS, whereas the remaining sulfatide markers were quantified using the C16:0-S IS (Table 3).

### 3.3. Data from the First Year of NBS for MLD

**Figure 4:**
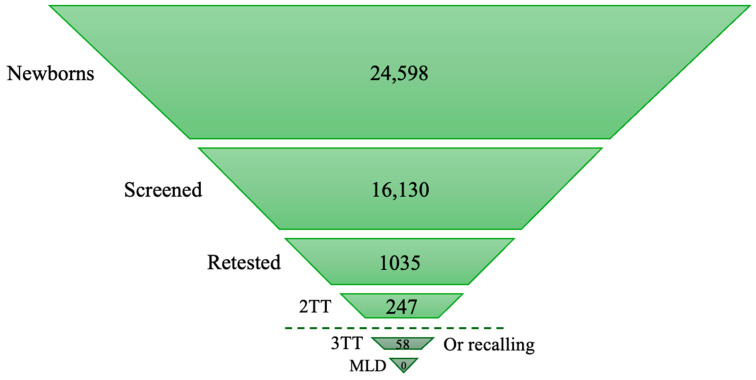
The funnel chart summarizes the tests performed on newborns screened since the beginning of the pilot project.

Between 15 July 2024 and 31 July 2025, a total of 24,598 full-term, full-body-weight neonates were born in the newborn centers participating in the MLD screening project. Of these eligible neonates, parental consent for participation in the MLD NBS was obtained for 16,130 infants. Among these, 1035 newborns (6.41%) required duplicate 1TT retesting, 247 newborns (1.53%) underwent duplicate 2TT, and 58 newborns (0.36%) were subjected to 3TT (Figure 4).

To date, no newborn with MLD has been identified through this screening program. However, variants potentially responsible for reduced ARSA enzymatic activity were identified in 33 newborns (0.20%) as reported in Table 4.

One subject (S32) was heterozygous for the pathogenic *ARSA* variant c.475C>T (p.Gln159*) (rs760271887) with a corresponding residual ARSA enzymatic activity of 40%. The remaining variants identified in the *ARSA* gene corresponded to those associated with PD-alleles. In particular, 17 subjects had the PD-allele c.[1055A>G;*96A>G], 15 in heterozygosity and 2 in homozygosity (S3; S21); 1 subject (S29) was found to be compound heterozygous for the PD-allele c.[1055A>G;*96A>G] and the c.1055A>G variant, and 13 newborns showed only the c.1055A>G variant, of which 9 were heterozygous and 4 were homozygous. The two variants, c.1055A>G (p.Asn352Ser) (rs2071421) and c.*96A>G (rs6151429), are in linkage disequilibrium and constitute the most common PD allele in European and North American populations. The polyadenylation site variant c.*96A>G appears to contribute most strongly to the low ARSA activity observed in pseudodeficiency. Homozygosity for the c.*96A>G variant (almost always on the same chromosome with c.1055A>G) is associated with ARSA activity that is approximately 10% of control values in synthetic-substrate-based assays and could result in diagnostic uncertainty [22,23].

Homozygosity for the c.1055A>G (p.Asn352Ser) variant alone results in 50% or more of the mean control ARSA activity in leukocytes [22,23].

Besides variants found in the *ARSA* gene, a pathogenic variant in the *SUMF1* gene was found in heterozygosity in one patient: c.191C>A (p.Ser64*) (rs1421066733).

### 3.4. Validation of the Screening Algorithm

To evaluate the robustness and internal validity of the proposed screening workflow, 1TT, 2TT and 3TT were assessed in MLD-affected subjects.

**Figure 5:**
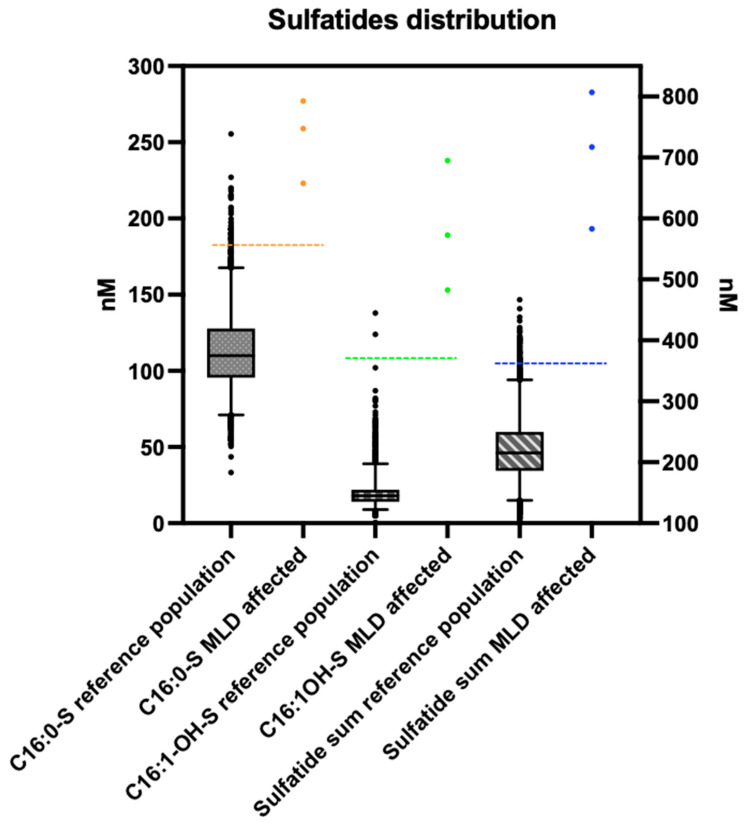
The graph displays box plots showing the distribution of C16:0-S concentrations (dotted box plots) in a reference population, together with data from three neonatal DBS from MLD-affected subjects (orange dots), plotted on the left *y*-axis. The second box plot refers to the distribution of C16:1-OH-S concentrations (aqua red box plots) in a reference population, together with data from three neonatal DBS from MLD-affected subjects (green dots), plotted on the left *y*-axis. It also shows the distribution of the sum of the four sulfatides (lined box plots) in the reference population, along with data from three neonatal DBS from MLD-affected subjects (blue dots), plotted on the right *y*-axis. Dotted horizontal lines indicate the cut-off values (orange and green lines refer to the left *y*-axis, while the blue line refers to the right *y*-axis).

Three Guthrie cards, stored at the Center of Newborn Screening, collected at 48–72 h of life and corresponding to patients later diagnosed at San Raffaele Hospital, were analyzed. These neonatal DBS, obtained prior to the implementation of the MLD screening protocol, provided a unique opportunity to assess sulfatide accumulation in newborn specimens. Despite the storage conditions, the measured levels of C16:0-S and the total sulfatide concentration (the primary interpretative criteria) were markedly above the updated cut-off values reported in Table 2 (Figure 5 and Table 5). These three samples were recalculated using both IS as a future perspective.

Regarding the 2TT, the three Guthrie cards from affected newborns were unusable due to storage at room temperature, which led to degradation of ARSA enzymatic activity.

**Figure 6:**
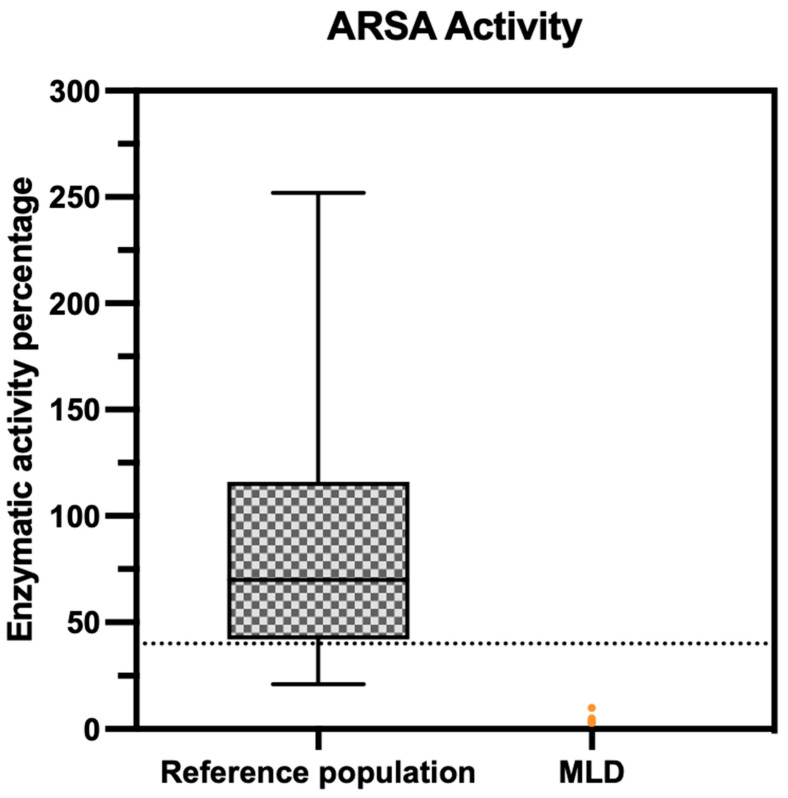
The graph shows ARSA enzymatic activity measured in DBS from the healthy reference population (n = 145, represented by the square boxplot) and in blood from MLD patients (n = 6, shown as orange dots) spotted on DBS. The dotted line represents the 20th percentile of the reference population.

Additionally, 6 DBS samples from MLD-affected patients (diagnosed by genetic testing) were analyzed. These samples were obtained from individuals older than the typical neonatal screening age. Blood from these subjects was collected via peripheral blood spotting onto DBS cards and processed using the same analytical workflow applied to neonatal DBS samples (Figure 6, Table 6).

## 4. Discussion

NBS for MLD offers the possibility of transforming the disease course by permitting the recognition of affected newborns at a stage when interventions such as Arsa-cel for early-onset forms or HSCT for later-onset forms are most effective.

In this study, we present the MLD NBS protocol and workflow in Lombardy (Italy), including the validation of the mass spectrometry-based analytical workflow, the set-up of a screening algorithm and the confirmation of its reliability.

A key achievement of the pilot phase was the development and consolidation of the mass spectrometry-based method for sulfatide determination. Over the first year of NBS, the assay demonstrated robust performance: quality control materials consistently remained within predefined limits, and the initially established cut-off for C16:0-S remained stable even after recalculation on a sample cohort four times larger than the one used at the start of the project.

Despite these strengths, several analytical limitations persist. A pure compound suitable for clinical validation is available exclusively for C16:0-S. This constrained availability of reference materials hampers accurate calibration and contributes to considerable inter-laboratory variability. Although the use of D_13_-C16:1OH-S improved the accuracy and precision in the quantification of the C16:1OH-S marker, a pure reference standard would be required for full assay standardization.

These constraints highlight the urgent need for expanded and standardized analytical materials to support wider implementation and harmonization of MLD NBS. Notably, when the reference population was recalculated (Table 2), percentile values shifted slightly even for markers whose analytical procedures had not been modified.

It is also important to note that the screening workflow implemented in our pilot study is not directly comparable with those adopted by other MLD screening centers [19,21,26]. Differences in percentile-based cutoffs and, critically, the use of a 3TT approach in our algorithm with the German approach [22,23], versus the two-tier strategies commonly used elsewhere, inevitably influence recall rates, analytical performance, and the interpretation of screening outputs. These methodological differences should therefore be considered when comparing detection rates or evaluating inter-center variability.

Our screening results showed that 6.41% of newborns underwent retesting. This relatively high proportion may be related, at least in part, to pre-analytical variability of the filter paper across different spots. Notably, when retesting was performed on double spots, the percentage of positive samples decreased to 1.53%.

The discrepancy between the initial retest rate (6.41%) and the proportion proceeding to 2TT (1.53%) is explained by the sequential confirmatory workflow. Samples exceeding the 97.5th percentile at first-tier screening were retested in duplicate using two independent DBS punches, and the mean value was used for interpretation. A stricter 99th percentile cut-off was subsequently applied to the averaged results to define eligibility for 2TT. This stepwise approach reduced the number of samples forwarded to confirmatory analysis.

The expanded dataset from the first year of the pilot program also allowed reassessment of the initial percentile-based cut-off strategy. The 97.5th percentile threshold was intentionally selected during the early implementation phase to maximize sensitivity and minimize the risk of false-negative results for a severe and rapidly progressive disorder such as MLD. However, the observed retest burden suggests that further optimization of the screening algorithm is warranted. Slightly more stringent percentile thresholds may improve the balance between sensitivity and specificity, thereby reducing unnecessary retesting while preserving clinical sensitivity.

In addition, the analytical method was validated exclusively for C16:0-S, while previous reports suggest that inclusion of C16:1-OH-S could potentially contribute to a reduction in false positive rates [27]. In this context, the adoption of C16:1OH-S as a decision marker could improve overall algorithm performance and further refine cut-off specificity. Nevertheless, due to the low number of screen-positive neonatal MLD cases, definitive optimization of percentile thresholds and biomarker combinations remains challenging. Additional longitudinal data, inclusion of confirmed positive samples, and inter-laboratory harmonization studies will be essential to establish the most clinically appropriate balance between sensitivity, specificity, and recall rate for large-scale implementation of MLD NBS.

Implementation of the ARSA 2TT was crucial to reduce false positives and prevent unnecessary recalls for families confronted with the suspicion of a severe rare disease. However, the assay is technically demanding, fully manual, and requires approximately two days to complete. It also necessitates highly skilled personnel and batching of samples to avoid reagent waste, which inevitably prolongs turnaround times. In high-throughput laboratory settings, maintaining a controlled temperature across all processing stages of the DBS card, crucial for preserving residual ARSA enzymatic activity, proves particularly challenging. These operational barriers severely affect assay reliability and contribute to the observed variability among different laboratories testing this NBS workflow.

In our cohort, ARSA 2TT revealed that 0.36% of screened neonates fell below the 20th percentile, encompassing *ARSA* and *SUMF1* heterozygotes, as well as *ARSA* PD-alleles carriers. While the primary goal of screening is early identification of affected neonates, inclusion of unaffected carriers during this pilot phase was critical for refining the algorithm and improving differential diagnosis.

Given these limitations, incorporating a genetic 3TT emerges as a fundamental component of an effective NBS algorithm for MLD. Consistent with European consensus-based recommendations [21], MLD NBS should not rely solely on the ARSA enzymatic assay. Genetic testing allows variant-level resolution of cases with low enzymatic activity or inconclusive biochemical results, improving specificity, avoiding unnecessary family recalls, and differentiating true MLD, *ARSA*-PD, and biochemically similar disorders such as those caused by *PSAP* or *SUMF1* mutations. This approach has been successfully implemented in Germany, where a three-tier strategy including genetic testing identified three confirmed MLD cases among 109,259 newborns [18].

In the absence of a genetic 3TT, 0.36% of newborns in our cohort would have required recall for a second DBS to verify potential positive findings. Among these, approximately 0.20% would likely have remained positive due to underlying genetic variants associated with reduced enzymatic activity, thereby necessitating referral to the appropriate clinical reference center for further evaluation.

Within our laboratory, WES with an in silico gene panel currently represents the most feasible approach for a genetic 3TT, as it is already integrated into the NBS workflow for inherited metabolic disorders. Genetic results have been obtained within 4–5 days, thereby reducing screening uncertainty and preventing family recall based solely on biochemical suspicion.

Future availability of a robust, standardized assay for C16:1OH-S might enable WES to be implemented directly as 2TT, bypassing enzymatic analysis, which remains analytically challenging to perform on DBS. WES would likely allow the detection of point mutations and small indels, which appear to account for the majority of cases (90–95%), whereas large rearrangements, currently reported in less than 1% of pathogenic variants [10], may require confirmation using more specialized techniques.

As an ultra-rare disease, MLD poses challenges for assay optimization and statistical validation due to the scarcity of neonatal positive samples. High participation rates in NBS programs are therefore crucial for generating sufficient data to refine test accuracy and characterize disease prevalence. Our participation rate compares favorably with those reported in other MLD NBS initiatives, including the program in Florence [19] and those recently published from the UK [19] and the US [21]. Direct comparison of recall rates, positive predictive values, and analytical performance underscores both the progress made and the areas where methodological harmonization remains necessary.

During the first year of screening, no MLD-affected newborns were identified. However, validation using confirmed MLD cases demonstrated that all exhibited sulfatide levels well above cut-offs and ARSA activity below the established threshold, confirming the discriminatory power of the algorithm. The scarcity of neonatal positive samples remains a key challenge for assay optimization and statistical validation, underscoring the importance of ongoing data collection and methodological refinement.

## 5. Conclusions

MLD is a progressive and potentially fatal disorder with a narrow pre-symptomatic therapeutic window during which gene therapy provides maximal clinical benefit. Consequently, pre-symptomatic identification of affected individuals through public health initiatives, such as NBS, is essential.

The first-year implementation of MLD NBS in Lombardy demonstrates that a mass spectrometry-based sulfatide assay, combined with a multi-tiered algorithm including ARSA enzymatic testing and genetic analysis, is feasible and reliable. While no affected newborns were identified, retrospective testing of MLD cases confirmed the algorithm’s discriminatory power. Key challenges remain, including limited availability of reference standards for sulfatide species beyond C16:0-S and the operational complexity of the enzymatic assay. Incorporation of a genetic 3TT and expansion of analytical validation, particularly including additional sulfatide species, are critical to improving specificity, reducing false positives, and enabling timely identification of affected infants.

## Figures and Tables

**Table 1:** Anamnestic and clinical characteristics of MLD patients included as positive controls.

Case	Date of Birth	Age at Sample Collection	Sex	Age at Onset	Treatment
1	16 October 2012	10 y10 m	M	10 y8 m	GT
2	19 March 2007	16 y6 m	F	15 y	-
3	7 April 2017	6 y9 m	F	3 y	-
4	23 December 2018	5 y2 m	M	2 y4 m	-
5	31 October 2015	8 y7 m	F	6 y10 m	T
6	26 April 1973	50 y10 m	M	35 y	-
7 *	7 December 2020	3 d	F	15 m	-
8 *	31 October 2021	2 d	M	-	GT
9 *	28 March 2022	2 d	F	15 m	-

* Newborn screening DBS. Y = years, M = months, D = days, GT = gene therapy, T = transplant. Treatments were initiated after sample collection.

**Table 2:** Percentile distribution of sulfatide concentrations in the screened newborn population.

Sulfatide	2.5th	25th	50th	75th	97.5th	99.0th
C16:0-S	66	91	106	123	160	167
C16:0-OH-S	19	30	38	49	72	76
C16:1-S	<1	3	4	5	10	11
C16:1-OH-S	9	14	18	22	31	33
Sum *	105	145	170	199	256	270

All concentrations are reported in nmol/L. * Sum refers to the sum of the 4 species of sulfatides.

**Table 3:** Updated percentile distribution of sulfatide concentrations in the screened newborn population analyzed with the new internal standard formula containing C16:0-S and C16:1-OH-S internal standards.

Sulfatide	2.5th	25th	50th	75th	97.5th	99.0th
C16:0-S	71	95	110	128	168	181
C16:0-OH-S	18	29	38	50	81	90
C16:1-S	2	3	4	5	8	9
C16:1-OH-S	36	51	60	72	101	112
Sum *	137	186	215	250	347	374

All concentrations are reported in nmol/L. * Sum refers to the sum of the 4 species of sulfatides.

**Table 4:** Biochemical and genetic results from 33 selected newborns who showed variants potentially responsible for reduced enzymatic activity. The two samples heterozygous for pathogenic variants are highlighted in green. n.a. means the procedure was not performed.

Sample	C16:0-S (nM)	ARSA Assay (%)	WES	
			Allele	Allele
S1	210	8	*ARSA:*c.1055A>G	
S2	191	12	*ARSA:*c.[1055A>G;*96A>G]	
S3	180	12	*ARSA:*c.[1055A>G;*96A>G]	*ARSA:*c.[1055A>G;*96A>G]
S4	222	15	*ARSA:*c.[1055A>G;*96A>G]	
S5	224	18	*ARSA:*c.1055A>G	*ARSA:*c.1055A>G
S6	186	19	*ARSA:*c.[1055A>G;*96A>G]	
S7	173	21	*ARSA:*c.1055A>G	
S8	n.a.	22	*ARSA:*c.[1055A>G;*96A>G]	
S9	188	23	*ARSA:*c.1055A>G	*ARSA:*c.1055A>G
S10	194	23	*ARSA:*c.[1055A>G;*96A>G]	
S11	178	24	*ARSA:*c.1055A>G	
S12	n.a.	24	*ARSA:*c.[1055A>G;*96A>G]	
S13	129	26	*ARSA:*c.1055A>G	*ARSA:*c.1055A>G
S14	218	27	*ARSA:*c.1055A>G	
S15	181	27	*ARSA:*c.1055A>G	
S16	190	28	*ARSA:*c.[1055A>G;*96A>G]	
S17	186	28	*ARSA:*c.[1055A>G;*96A>G]	
S18	214	29	*ARSA:*c.1055A>G	
S19	188	30	*ARSA:*c.1055A>G	*ARSA:*c.1055A>G
S20	176	31	*ARSA:*c.[1055A>G;*96A>G]	
S21	146	33	*ARSA:*c.[1055A>G;*96A>G]	*ARSA:*c.[1055A>G;*96A>G]
S22	175	33	*SUMF1:*c.191C>A	
S23	207	33	*ARSA:*c.1055A>G	
S24	154	34	*ARSA:*c.[1055A>G;*96A>G]	
S25	147	35	*ARSA:*c.1055A>G	
S26	176	35	*ARSA:*c.[1055A>G;*96A>G]	
S27	131	35	*ARSA:*c.[1055A>G;*96A>G]	
S28	171	37	*ARSA:*c.[1055A>G;*96A>G]	
S29	166	37	*ARSA:*c.[1055A>G;*96A>G]	*ARSA:*c.1055A>G
S30	206	38	*ARSA:*c.[1055A>G;*96A>G]	
S31	164	38	*ARSA:*c.1055A>G	
S32	162	40	*ARSA:*c.[1055A>G;*96A>G]	
S33	177	40	*ARSA:*c.475C>T	

**Table 5:** Biochemical and genetic results of newborn screening Guthrie cards from 3 MLD-affected subjects. n.a. is an acronym indicating that the procedure was not performed.

Test	Analytes	Case 1	Case 2	Case 3
1TT	C16:0 (nM)	259	223	277
	C16:1 (nM)	18	28	27
	C16:0-OH (nM)	250	179	265
	C16:1-OH (nM)	189	153	238
	Sum (nM)	717	583	807
3TT	Variant 1	c.465+1G>A	c.1283C>T	c.465+1G>A
	Variant 2		c.526C>T	c.1108-3C>G

**Table 6:** ARSA activity and genetic results of Guthrie card spots from MLD-affected subjects older than the typical neonatal screening age.

Test	Analytes	Case 1	Case 2	Case 3	Case 4	Case 5	Case 6
2TT	ARSA (%)	5	3	3	10	4	3
3TT	Variant 1	c.185_186dupCA	c.352delT	c.256C>T	c.465+1G>A	c.465+1G>A	c.608A>G
	Variant 2	c.514G>T	c.869G>T	c.1174C>T	c.1471T>G	c.542T>G	c.418dupC

## Data Availability

Data are unavailable due to privacy.

## Abbreviations

The following abbreviations are used in this manuscript:

MLD	Metachromatic leukodystrophy
ARSA	Arylsulfatase A
NBS	Newborn screening
1TT	First-tier test
2TT	Second-tier test
3TT	Third-tier test
ARSA-PD	Pseudodeficiency allele
HSCT	Hematopoietic stem cell transplantation
DBS	Dried blood spot
UPLC-MS/MS	Ultra-pressure liquid chromatography tandem mass spectrometry
MRM	Multiple reaction monitoring
C16:0-S	C16:0-Sulfatide
C16:0-OH-S	C16:0-OH-Sulfatide
C16:1-OH-S	C16:1-OH-Sulfatide
C16:1-S	C16:1-Sulfatide
IS	Internal standard
D_5_-C16:0-S	C16:0-S deuterium marked
D_13_-C16:1-OH	C16:1-OH-S deuterium marked
LOD	Limit of detection
LOQ	Limit of quantification
LOB	Limit of Blank
WES	Whole exome sequencing
